# Microarray Profiling and Co-Expression Network Analysis of LncRNAs and mRNAs in Acute Respiratory Distress Syndrome Mouse Model

**DOI:** 10.3390/pathogens11050532

**Published:** 2022-05-02

**Authors:** Xiaoling Wu, Chenjie Ma, Qinmei Ma, Peipei Zhuang, Guangcun Deng

**Affiliations:** 1 Key Laboratory of Ministry of Education for Conservation and Utilization of Special Biological Resources in the Western China, Ningxia University, Yinchuan 750021, China; wuxiaol@nxu.edu.cn (X.W.); machj@nxu.edu.cn (C.M.); 18195180646@163.com (Q.M.); zhuangpeipei890120@163.com (P.Z.); 2 School of Life Science, Ningxia University, Yinchuan 750021, China

**Keywords:** LncRNAs, mRNA, microarray, pathogen, ARDS

## Abstract

Background: Long noncoding RNAs (LncRNAs) play critical roles in many respiratory diseases. Acute respiratory distress syndrome (ARDS) is a destructive clinical syndrome of respiratory diseases. However, the potential mechanism of LncRNAs on ARDS remains largely unknown. Methods: To identify the profiles of LncRNAs and mRNAs in the LPS-induced ARDS mouse model, the microarray analyses were hired to detect the expression of LncRNAs and mRNAs in present study. Subsequently, microarray data were verified by quantitative qRT-PCR. Functional annotation on DE mRNAs and LncRNAs were carried out by bioinformatics analysis. Furthermore, the role of selected DE LncRNAs on correlated genes was confirmed by si-RNA and Western blot. Results: The expression of 2110 LncRNAs and 2690 mRNAs were significantly changed, which were further confirmed by qRT-PCR. GO and KEGG analysis indicated that the up-regulated mRNAs were mainly related to a defense response and tumor necrosis factor (TNF) signaling pathway, respectively. LncRNA-mRNA co-expression analyses showed that LncRNAs NR_003508, ENSMUST00000131638, ENSMUST00000119467, and ENSMUST00000124853 may correlate to MLKL, RIPK3, RIPK1, Caspase1, and NLRP3, respectively, or cooperatively, which were highly involved in the cell necroptosis process. Furthermore, siRNA for NR_003508 confirmed the co-expression analyses results. Conclusion: To summarize, this study implied that the DE LncRNAs could be potent regulators and target genes of ARDS and will provide a novel insight into the regulation of the pathogenesis of ARDS.

## 1. Introduction

As the severe form of acute lung injury (ALI), acute respiratory distress syndrome (ARDS) is a process of nonhydrostatic pulmonary edema, bilateral normal cardiac filling pressures, and acute hypoxemic respiratory failure [1,2]. Although improvement in supportive care, ARDS are burdened by high mortality, being responsible for 30% to 40% of the deaths globally [3]. Of note, the pathogenesis of ARDS is an uncontrolled inflammatory response, which lead to the accumulation of alveolar macrophages and neutrophils in the lung and accompanied by diffuse alveolar damage and pulmonary edema [4]. Thus far, the pathogenesis of ALI needs to be elucidated. Increasingly evidence suggested that trauma, severe sepsis, and inhaling harmful gas can cause ALI/ARDS; however, the infection of bacteria and virus is one of the key reasons of ALI/ARDS by regulating the genes expression [5,6]. In particular, the latest study showed that approximately 15% to 30% of patients infected with the COVID-19 virus developed ARDS [7]. It is also confirmed that pathological findings of the COVID-19 were associated with acute respiratory distress syndrome [8]. Intriguingly, accumulating evidence has shown that LncRNA could manage gene expression and serve as key regulator in ALI/ARDS [9]. Therefore, it is urgent to identify specific LncRNA being a potential therapeutic target for pathogen-induced ARDS.

Long non-coding RNA (LncRNA) is an RNA molecule longer than 200 nucleotides, and they have emerged as important regulators of gene expression in diverse biological processes [10]. An increasing body of study indicates that LncRNA is involved in cell apoptosis, proliferation and differentiation, and a variety of diseases, such as tumors and infectious diseases [11,12,13]. To further explore the pathogenesis mechanism of ARDS, it is worth investigating the expression profiles of LncRNAs in mouse lungs infected by lipopolysaccharides (LPS). Cell death, such as apoptosis and necrosis, has been considered a potential mechanism in ALI/ARDS and multiorgan dysfunction syndrome [14]. Accordingly, it is also a challenge to narrow down the group of candidate mRNAs associated with cell death that are potentially responsible for the ARDS.

With this in mind, a microarray analysis was used to interrogate the profiles of differentially expressed mRNA and LncRNA in ARDS mice. Meanwhile, Kyoto Encyclopedia of Genes and Genomes (KEGG) pathway and Gene Ontology (GO) categories enrichment analyses were utilized to explore the unique functions of LncRNAs and mRNA. LncRNA-mRNA co-expression network was further performed to detect them mutual regulation. Furthermore, siRNA for selected LncRNA was used to confirm the co-expression result. Taken together, this study focused on this aspect may bring new insights into the mechanism of ALI.

## 2. Results

### 2.1. Establishment and Assessment of the ARDS Model

**Figure 1:**
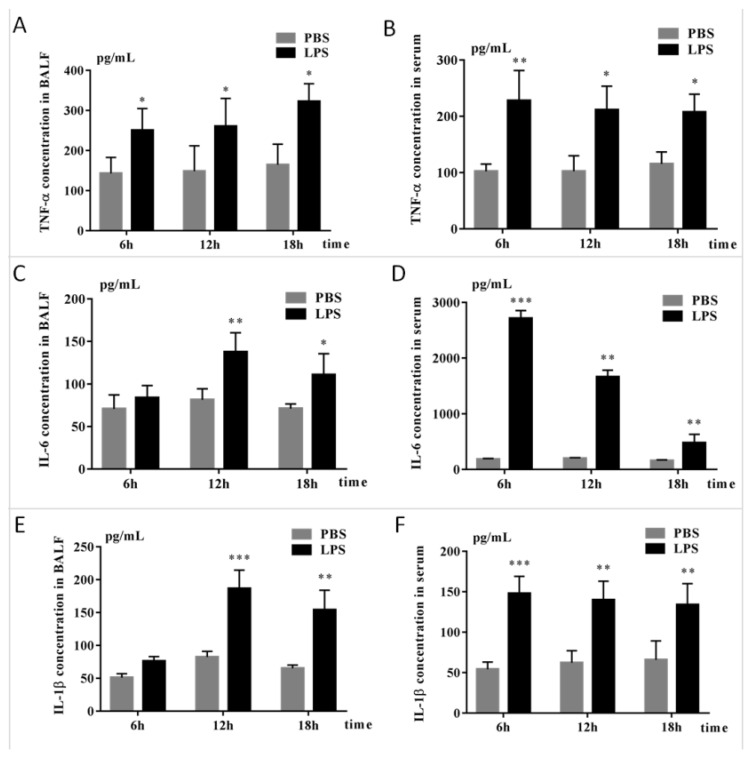
The inflammation factor of BALF and serum in LPS-induced mice. ELISA was used to detect the TNF-α (**A**,**B**), IL-6 (**C**,**D**), and IL-1β (**E**,**F**) of bronchoalveolar lavage fluid (BALF) and serum collected from mice treated with LPS and PBS at different time points. The data displayed in histograms are expressed as means ± standard deviation. * *p* < 0.05; ** *p* < 0.01; *** *p* < 0.001 compared with the PBS group. Compared to a naïve control, * *p* < 0.05; ** *p* < 0.01. Three independent, triplicated experiments were performed.

**Figure 2:**
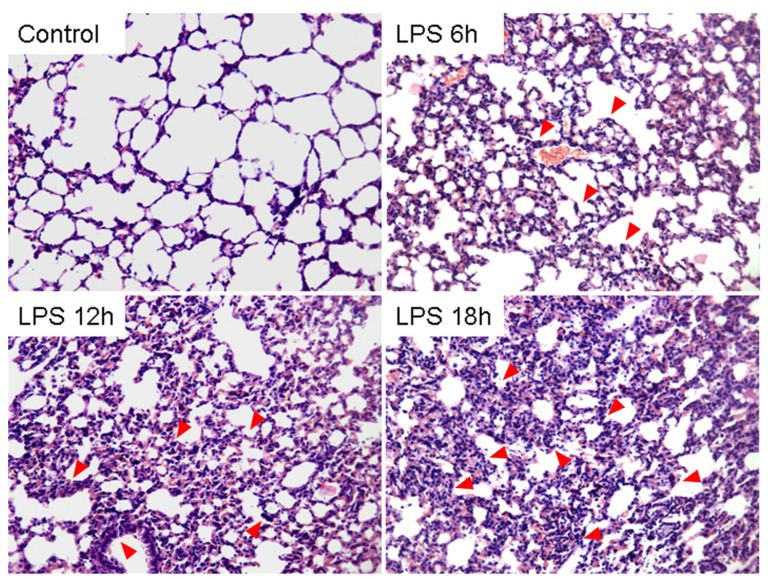
Histopathology of LPS-induced acute lung injury in mice lung (HE staining, 200×). The histological patterns of the lung. Acute lung injury was induced by lipopolysaccharides via intraperitoneal injection for 6 h, 12 h, and 18 h. Subsequently, histological examinations were measured by HE staining. (inflammatory infiltration, red arrowhead pointing; ×200, scale bars, 100 μm). Three independent, triplicated experiments were performed.

ARDS is characterized by increased pulmonary microvascular permeability, histologic features of pulmonary parenchymal injury, and alveolar-capillary endothelial cell and inflammation factor [15].To assess the ARDS model, the inflammation factor of mice induced by LPS at different times was detected. As shown in Figure 1, TNF-α, IL-6, and IL-1β in serum and bronchoalveolar lavage fluid (BALF) of mice were significantly elevated in LPS-challenged mice compared with the PBS group. Consistent with expectation, histopathology of mice lungs injected with LPS also showed exudation and deposits of interstitial neutrophils and neutrophilic infiltration present in the alveolar septa and lumens of the ARDS model (Figure 2). The above results suggested that LPS-induced ARDS mouse model has been successfully established.

### 2.2. Differentially Expressed LncRNA and mRNAs in ALI

**Figure 3:**
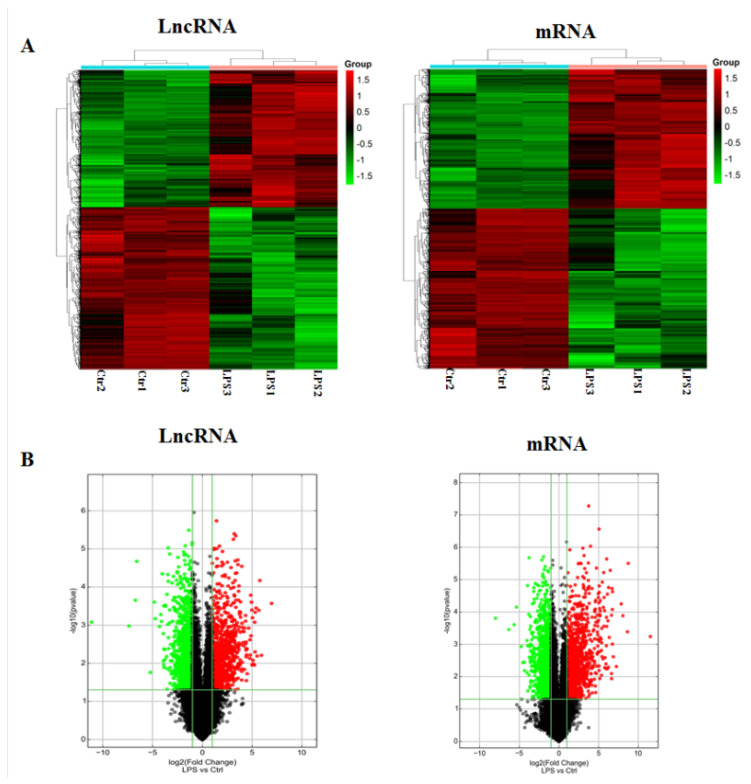
Heatmap and volcano plots showing LncRNA and mRNA levels. (**A**) Screening criteria were as follows: *p* ≤ 0.05 for LncRNAs and mRNAs. Expression values are described in terms of a color scale; the colors from green to red indicated the intensity expression. Each column represents one sample, and each row represents a transcript. (**B**) Volcano plots reflect the number, significance, and reliability of differentially expressed LncRNA and mRNAs. The abscissa is log2 (fold change), and the ordinate is −log10 (*p*−value). Red dots are up−regulated genes. Green dots are down-regulated. Black dots indicate that the two sets of genes are identical.

We used the Arraystar Mouse LncRNA Array v2.0 to identify differentially expressed LncRNA and mRNAs in the mouse genome. Based on the threshold of fold-change ≥ 2.0 and *p*-value ≤ 0.05, LncRNAs were collected from the authoritative data sources, including Ref Seq (https://www.ncbi.nlm.nih.gov/Refseq/, accessed on 8 November 2018), UCSC Known genes (https://genome.ucsc.edu/, accessed on 8 November 2018), Ensembl (http://asia.ensembl.org/index.html, accessed on 8 November 2018), fantom3 (http://fantom3.gsc.riken.jp/, accessed on 8 November 2018), RNAdb (http://research.imb.uq.edu.au/rnadb, accessed on 8 November 2018), UCR (http://users.soe.ucsc.edu/~jill/ultra.html, accessed on 8 November 2018), and NRED (https://www.hsls.pitt.edu/obrc/index.php?page=URL1237993821, accessed on 8 November 2018).Hierarchical clustering (Figure 3A) and volcano plots (Figure 3B) showed systematic variations expression of LncRNAs and mRNAs from lung tissues of mice treated with LPS or PBS. Compared with control group, 2110 LncRNAs and 2690 mRNAs were differentially expressed in the LPS treatment group. Of these, a total of 967 LncRNAs and 1252 mRNAs were significantly up-regulated, while 1143 LncRNAs and 1438 mRNAs were down-regulated (fold change ≥ 2.0, Tables S1 and S2).

### 2.3. Verification of LncRNAs and mRNAs by qRT-PCR

**Figure 4:**
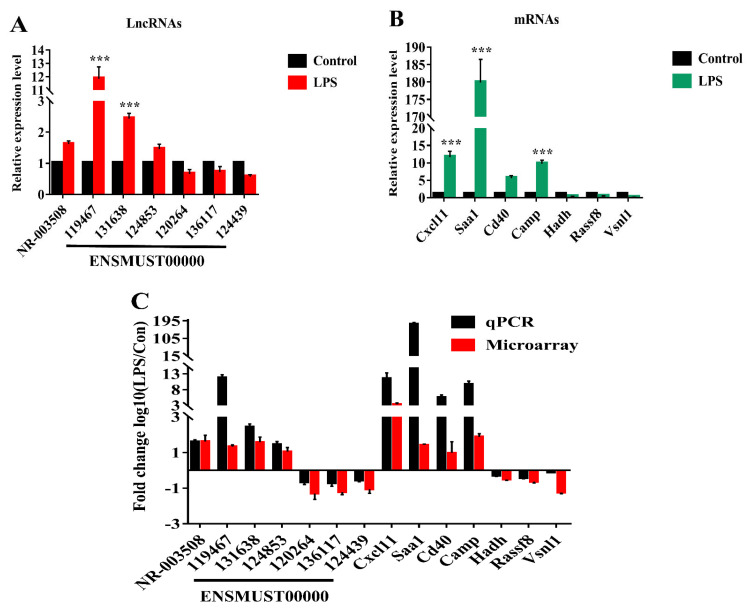
Validation of microarray data by qRT-PCR. (**A**) 7 LncRNA and (**B**) 7 DE mRNAs between LPS and PBS groups were validated by qRT-PCR. The relative expression of RNA was normalized to β-actin. The data displayed in histograms are expressed as means ± standard deviation. *** *p* < 0.001 compared with the control group. (**C**) Microarray data and qRT-PCR results. The heights of the columns represent the fold changes (log10 transformed).

Seven candidate LncRNAs and seven mRNAs were identified by qRT-PCR in mice treated with LPS/PBS for 12 h. As shown in Figure 4A, compared with the control, the selected seven LncRNAs of the LPS group were significantly differentially expressed. The expression of NR_003508, ENSMUST00000119467, ENSMUST00000131638, and ENSMUST00000124853 were up-regulated, while ENSMUST00000120264, ENSMUST00000136117, and ENSMUST00000124439 were down-regulated, and their associated genes have previously been reported to be involved in anti-virus and immune activation and cell programmed death. For instance, the NR_003508-associated gene is MX2, which is an interferon-induced inhibitor of HIV-1 infection [16]. The ENSMUST00000119467-associated gene is Gm12250, an immune activation signature [17]. In addition, seven mRNAs were demonstrated by qRT-PCR. The consistent results between qRT-PCR and microarray data were shown in Figure 4C.

### 2.4. GO and KEGG Enrichment Analysis of DE LncRNA Target Genes and DE mRNAs

The Gene Ontology Project provides a controlled vocabulary to describe the properties of genes and gene products in any organism (http://www.geneontology.org, accessed on 8 November 2018). Because no complete annotation database is available for categorizing LncRNA, the ontology generally includes three areas: cellular component, molecular function, and biological process. In Bioconductor’s top GO, Fisher’s exact test was used to discover whether there was more overlap between DE listings and GO annotations.

**Figure 5:**
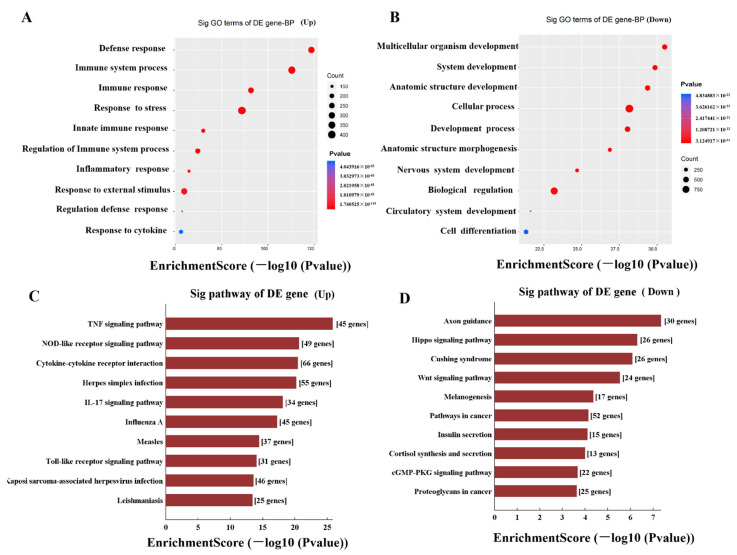
GO enrichment and KEGG pathway analysis of differentially expressed genes in LPS-induced mice. (**A**) GO annotation of up-regulated mRNAs, with the top 10 functional GO terms. (**B**) GO annotation of down-regulated mRNAs, with the top 10 functional GO terms. (**C**) The top 10 pathways enriched among the up-regulated mRNAs in LPS challenged mice. (**D**) Significant pathways of downregulated mRNAs. The bar plot depicts the enrichment scores.

Accumulating evidence has shown that the expression of overlapping protein-coding genes or adjacent were regulated LncRNAs [16]. In our study, GO analyses were performed on DE mRNAs. The top 10 statistically significant GO terms of up-regulation and down-regulation are shown in Figure 5A,B, respectively. The results showed that the highest enriched GO terms targeted by up-regulated transcripts of biological processes included defense response, immune response, immune system process, response to stress, and innate immune response. The highest enriched GO terms targeted by down-regulated transcripts included multicellular organism development, system development, anatomic structure development, cellular process, development process, and anatomic structure morphogenesis. Moreover, based on the latest KEGG (http://www.genome.jp/kegg, accessed on 8 November 2018) database, pathway analysis of differentially expressed mRNAs is shown in Figure 5C,D. The up-regulated target genes were involved in the cytokine-cytokine receptor interaction, NOD-like receptor (NLR) signaling pathway, TNF-signaling pathway, etc. The down-regulated target genes were involved in Axon guidance and the Hippo signaling pathway. The *p*-value denotes the significance of the pathway. (The *p*-value cut-off is <0.05).

### 2.5. Co-Expression Correlations between mRNAs and LncRNAs

**Figure 6:**
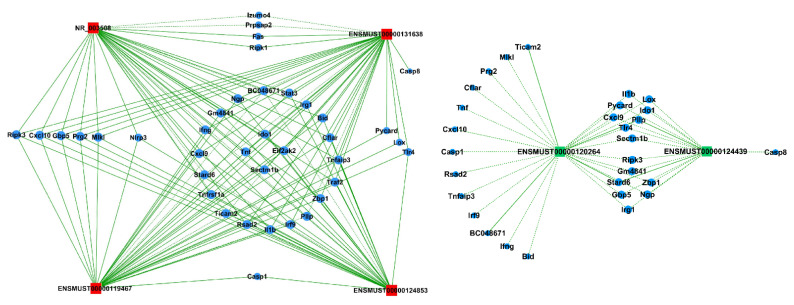
LncRNA-mRNA co-expression network in the “necroptosis” pathway. Nodes represent LncRNA, and circles represent coding genes. Red indicates up-regulated genes, and green indicates down-regulated genes. Solid lines and dashed lines represent positive correlations and negative correlations, respectively. Six LncRNAs were interacting with thirty-eight mRNAs in the meaningful “necroptosis” pathway.

Previous studies showed that there are some alternative pathways for cell death in acute lung injury, and LPS-induced acute lung injury is related to apoptosis via Fas/Fas ligand mechanisms [3]. Therefore, to predict whether the validated LncRNAs are involved in the cell death process, we selected six LncRNAs and built the network with mRNAs belonging to the meaningful “necroptosis” pathway (Figure 6). LncRNA-mRNA co-expression networks were constructed by using Cytoscape software (the Cytoscape Consortium, San Diego, CA, USA) based on the normalized signal intensities of mRNA and LncRNA-specific expression levels. For further study, we used Pearson’s correlation coefficient ≥ 0.9 to identify LncRNAs and mRNAs. The co-expression network showed that LncRNAs NR_003508, ENSMUST00000131638, ENSMUST00000119467, and ENSMUST00000124853 positively corresponded to MLKL, RIPK3, and NLRP3, which were involved in the cell necrosis and pyroptosis process [18,19,20]. On the contrary, ENSMUST00000120264 and ENSMUST00000124439 were negatively related to RIPK3 and MLKL. Both NR_003508 and ENSMUST00000131638 interacted with RIPK1 and Fas, while ENSMUST00000119467 and ENSMUST00000124853 interacted with Caspase1. The results suggested that the interaction between LncRNAs and mRNAs is involved in the development of ARDS by regulating the process of cell death.

### 2.6. si-RNA Targeting NR_003508 Decreased Cell Necroptosis in RAW264.7 Cell Induced by LPS

**Figure 7:**
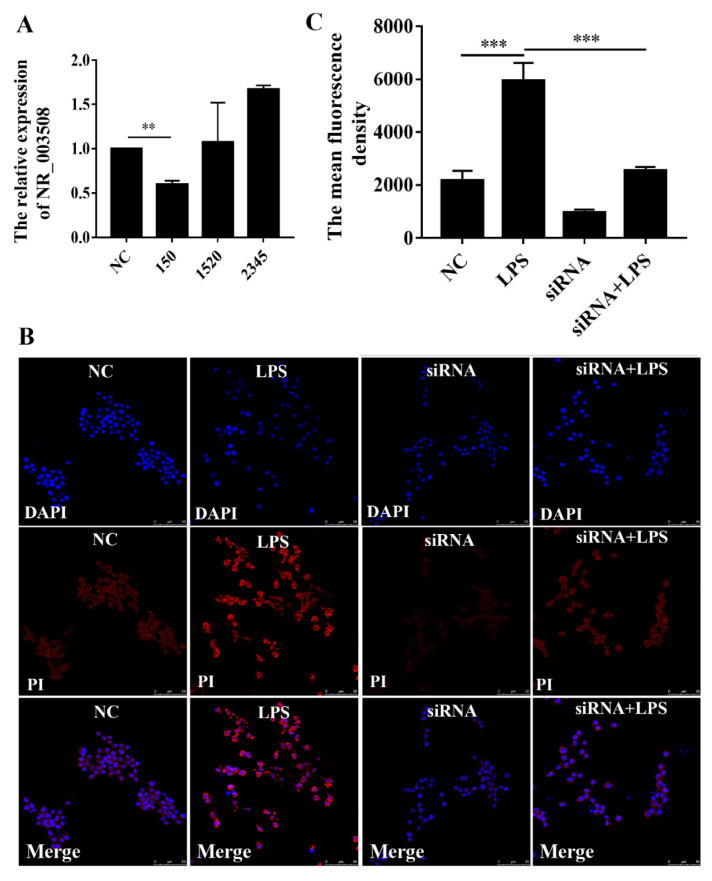
si-RNA for NR_003508 decreased the cell necrosis in RAW264.7 cells. (**A**) To confirm the silencing efficiency of NR_003508, three kinds of siRNAs were selected and transfected into RAW264.7. The expression of NR_003508 was observed by qRT-PCR. ** *p* < 0.01, *** *p* < 0.001, compared with the control group. (**B**) Immunofluorescence was used to examine the effect of NR_003508 knockdown on cell necrosis with LPS stimulation for 12 h. (**C**) Ten cells per group were picked randomly, and the mean fluorescence density was calculated by Image J. Three independent, triplicated experiments were performed.

**Figure 8:**
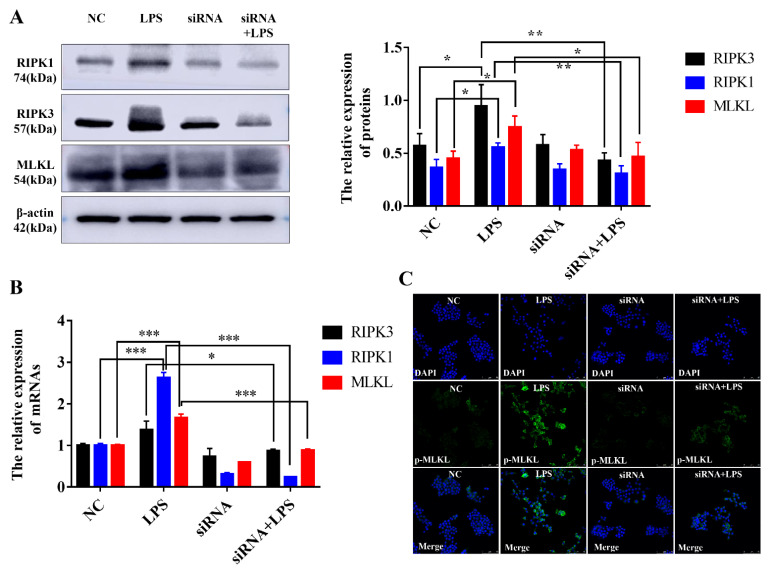
si-RNA for NR_003508 decreased the necroptosis-associated genes expression in RAW264.7 cells. (**A**) The representative immunoblotting image of RIPK1, RIPK3, and MLKL in the presence or absence of si-NR_003508 transfection in the RAW264.7 cells with LPS stimulation. (**B**) qRT-PCR was used to detect RIPK1, RIPK3, and MLKL in RAW264.7 cells with LPS stimulation for 12 h. (**C**) The expression of p-MLKL was observed by immunofluorescence. * *p* < 0.05, ** *p* < 0.01,*** *p* < 0.001.Three independent experiments were performed.

Following with co-expression network, we used siRNAs for NR_003508 to validate the effects on the “necroptosis” pathway in RAW264.7 cells. Since NR_003508 was up-regulated after LPS stimulation, we designed and synthesized the si-RNA of NR_003508. As shown in Figure 7A, #150 siRNA effectively reduced the expression of NR_003508. Subsequently, PI dye was utilized to examine the effect of NR_003508 on necrosis of RAW264.7 cells with LPS stimulation. Compared with LPS group, the mean fluorescence density significantly decreased in siRNA + LPS group, which indicated siRNA targeting NR-003508 could inhibit LPS-induced necrosis (Figure 7B,C). Recent studies suggest that necroptosis is mediated by RIPK3. Depletion of RIP3 abolished necroptosis. Of note, RIPK3 is a key gene of necrosis that formed necrosome with RIPK1. Moreover, RIPK3 phosphorylated MLKL at the threonine 357 and serine 358 residues, and these phosphorylation events are critical for necroptosis [21]. As shown in Figure 8A,B, si-NR_003508 down-regulated LPS-induced RIPK1, RIPK3, MLKL protein, and mRNA expression, which was confirmed to be involved in necroptosis. Immunofluorescence was used to examine the effect of knockdown of NR_003508 on LPS-induced expression of p-MLKL. As shown in Figure 8C, LPS significantly increased the expression of p-MLKL, but si-NR_003508 reversed this result. It indicated that NR_003508 also regulated the cell death process since NR_003508 decreased the necroptosis.

## 3. Discussion

As a grievous issue in critical care medicine, acute respiratory distress syndrome (ARDS) causes high rates of morbidity and mortality [22]. Of note, ARDS is usually caused by many factors such as surgery and pathogens infection. However, the underlying mechanisms of this process remains unclear. According to a previous study, LncRNAs play vital roles in diverse biological and immunological processes of infectious diseases [23]. In particular, pathogen infection will regulate LncRNAs expression, and some LncRNAs also are involved in disease processes, such as ALI. Thus, screening the specific LncRNA and mRNA associated with ARDS will be a novel insight for exploring new target drugs of pathogens.

LPS has been extensively considered as an ideal pharmacological research model of ALI followed by activating neutrophils and excessive generation of chemokines, such as alveolar TNF-α, a key mediator of alveolar inflammation in ARDS [24]. We then used LPS to establish an ARDS/ALI mice model. Inconsistent with previous studies, significant pathologic changes in lung tissue occurred in the LPS group as well as the accumulation of inflammation cytokines, including IL-1β, IL-6, and TNF-α. Of note, inflammation is essential for host defense, but aberrant inflammation contributes to the development of inflammatory diseases such as ARDS. Moreover, excessive cytokine secretions of ALI represent a state that involves inflammation of multiple organs [25]. Histopathology results also confirmed the results of the mice ARDS model with interstitial infiltration of neutrophils. Together with our findings, the results indicated that the LPS-induced ALI mouse model was established successfully.

Subsequently, we investigated LncRNA expression profiles of the ARDS model in mice; LPS could regulate 967 LncRNAs up and 1143 LncRNAs down by ≥ 2 fold. Of DE mRNAs, 1252 mRNAs were up-regulated, and 1438 mRNAs were down-regulated. In addition, volcano plots also demonstrated differences between LPS and control groups. To further confirm the results of microarray, selected LncRNAs and mRNAs were measured by qRT-PCR in mice treated with LPS/PBS. As expected, the qRT-PCR results of the selected mRNAs and LncRNAs were consistent with those of microarray (Figure 4C). Although our study found that LncRNAs and mRNAs expression in mice lung tissue induced by LPS were changed significantly, their biological functions are still obscure.

According to KEGG analysis, the up-regulated target genes induced by LPS were mainly involved in the NOD-like receptor signaling (NLRs) pathway, TNF-signaling pathway, and cytokine–cytokine receptor interaction. Our data were strongly supported by previous studies [26]. Among the molecules of the above signaling pathway, TNF-α, a critical inflammatory cytokine to be involved in cell necrosis and apoptosis, has long been implicated in ARDS pathology. Cell necrosis and apoptosis have been proven to be involved in the pathogenesis of ALI/ARDS [14]. Previous studies reported that many LncRNAs and associated genes are involved in stimulating oxidative stress during the pathogenesis of ALI/ARDS following cell death of the lung [27].

Based on the DE LncRNA and KEGG analysis results and previous studies, we hypothesized that DE LncRNAs should be involved in regulating the cell death signaling pathway in the ALI process. With this in mind, we selected six DE LncRNAs and built a LncRNA-mRNA co-expression network with DE mRNA of the “necroptosis” signal pathway. The LncRNA-mRNA co-expression network showed that different LncRNAs were differentially correlated with the same genes. Notably, LncRNA NR_003508, ENSMUST00000131638, ENSMUST00000119467, and ENSMUST00000124853 synergistically corresponded to MLKL and RIPK3. However, RIPK1, Caspase1, and NLRP3 were regulated by NR_003508, ENSMUST00000131638, ENSMUST00000119467, and ENSMUST00000124853, respectively, or together (As shown in Figure 6). On the contrary, ENSMUST00000120264 and ENSMUST00000124439 negatively regulated part of the above mRNAs. Our study implies that LncRNA is involved in ARDS development through the cell death signal pathway. Then, to confirm the reproducibility of LncRNA-mRNA co-expression network, we tested it by using Teng’s dada of ALI model [28]. As shown in Table S3, 69 mRNA and 1 LncRNA (ENSMUST00000124853) in Teng’s research were consistent with our LncRNA-mRNA co-expression network. Compared with our co-expression network, the coincidence rate of mRNA and LncRNA in Teng’s research were 86.43% and 16.67%, respectively. Moreover, 3 LncRNAs (uc007pnu.1, uc007rpk.1, and uc007rlu.1) in our database were found in the top 20 LncRNAs that were published by Juan Wang [26]. Despite the co-expression, we did not identify 100% resemblance with this research, which may have been caused by some complicated reasons, such as selection criteria, research interests, and so on; we hope our study will provide some data support for exploring the mechanism of ARDs.

A recent study indicated that at the cellular level, VILI induces necrotic cell death, and RIPK3 and mixed-lineage kinase domain-like pseudokinase (MLKL) contributed to necroptosis [18]. Intriguingly, RIPK1, a protein kinase that serves as a key regulator of life and death in TNF-exposed cells, remarkably mediates intestinal crypt apoptosis during chronic NF-κB activation [29]. Besides, NLRP3 and Caspase1 are involved in pyroptosis, a special cell death induced by inflammation. Interestingly, NLRP3 facilitates necrosis and Caspase1 activation by inflammasome-dependent and -independent pathways [30]. To verify our hypothesis, we used si-RNA for NR_003508 on proteins of the cell necroptosis pathway. Our data confirmed that si-RNA targeting NR_003508 suppressed the expression of RIPK1, RIPK3, and MLKL in RAW264.7 cells stimulated with LPS. Together with our findings, it suggested that LncRNA NR_003508, ENSMUST00000131638, ENSMUST00000119467, and ENSMUST00000124853 were involved in the ARDS process by positively regulating the cell necrosis or pyroptosis. These findings exemplify new insight into the potential role of mRNAs and LncRNAs in ARDS. However, the mechanisms and biological functions of LncRNAs in the pathogenesis of ARDS remain to be further explored.

## 4. Materials and Methods

### 4.1. Experimental Protocols for Acute Lung Injury Models

Male Kunming mice (20 ± 5 g) were purchased from the Center of Experimental Animals of Ningxia Medical University, Ningxia, China. All the animal experiments procedures were approved by Institutional Animal Care and Use Committee at Ningxia University. Mice were randomly divided into four groups with 12 mice per group (*n* = 48). (1) control group: mice were intravenously injected through the tail vein, with 50 μL of sterile PBS. (2) LPS 6 h group,12 h group, and 18 h group: mice were injected intravenously with 50 μL LPS from Escherichiacoli (serotype O55:B5; Sigma-Aldrich, St. Louis, MO, USA) for the indicated time, respectively (5 mg/kg). Then, mice were euthanized in a CO_2_ chamber. Subsequently, a median sternotomy was performed, and lungs were harvested after blood collection for serum. At the same time, the left lung was immediately placed in liquid nitrogen and processed for RNA extraction, as described below. The other lobes were prepared for histological examination.

### 4.2. Bronchoalveolar Lavage Fluid (BALF) Collection and Analysis

The mice were treated with LPS for 6, 12, and 18 h through tail vein injection, respectively. Then, mice were euthanized in a CO_2_ chamber. Lungs were gently lavaged through the tracheal tube 3 times with a total volume of 1 mL PBS. The collected BALF was immediately centrifuged at 1000 rpm for 5 min, and the supernatant was collected and stored at −80 °C. The levels of cytokines were detected by ELISA, and the inflammatory infiltration of neutrophils was detected by HE staining.

### 4.3. Enzyme-Linked Immunosorbent Assay (ELISA) for Mouse Interleukin-6 (IL-6), Interleukin-1β (IL-1β), and Tumor Necrosis Factor-Alpha (TNF-α)

To determine the IL-6, IL-1β, and TNF-α levels in the serum and BALF, we collected serum and BALF and stored them at −80 °C before analysis. Mouse TNF-α, IL-6, and IL-1β levels in the serum and BALF were determined by ELISA kits following the manufacturer’s instructions (NeoBioscience Technology Company Limited, Shenzhen, China).

### 4.4. Histopathological Evaluation

Next, 4% paraformaldehyde was used to fix mice lungs for 24 h. The lung tissue near bronchus were cut out and embedded in paraffin (5 mm thick). Finally, the tissue sections were stained with hematoxylin and eosin (HE) and visualized by optical microscope.

### 4.5. An Analysis of Differentially Expressed LncRNAs and mRNAs in ALI

RNA quality and integrity of each sample were assessed by NanoDrop ND-1000 and agarose gel electrophoresis, respectively. The Agilent Array platform was employed to perform microarray analysis. Sample preparation and hybridization were performed according to the manufacturer’s protocol. Briefly, rRNA was removed from total mRNA (mRNA-only™ Eukaryotic mRNA Isolation Kit, Epicenter, Madison, WI, USA). Then, samples was amplified and transcribed into fluorescent cRNA without 3’ bias by using a random prime, and labeled cRNAs were hybridized to microarray (8 × 60 K, Arraystar, Rockville, MD, USA,). After washing, the slides was scanned into images by an Agilent G2505C scanner and were analyzed by Agilent Feature Extraction software (version 11.0.1.1). The GeneSpring GX v11.5.1 software package (Agilent Technologies, Santa Clara, CA, USA) was used to perform quantile normalization and subsequent data processing. LncRNA, which has flags in present or marginal at least 3 out of 6 samples (“All Targets Value”), were chosen for further research. Differentially expressed LncRNAs between two groups were screened through fold change filtering.

### 4.6. Purification of Total RNA from the Male Kunming Mouse Tissue and RAW264.7 Cells

Total RNA was extracted from mouse tissues and RAW264.7 cells using TRIzol reagent, respectively. Briefly, Raw264.7 cells or 100 g mouse tissue were mixed with 1 mL of reagent and gently homogenized for 5 min. The homogenate in the aqueous and organic phases was separated with 200 ul of chloroform. Raw264.7 cells or mouse tissues were precipitated with 500 μL isopropanol, cleaned with 75% ethanol, and quantified by NanoDrop ND-1000 spectrophotometer.

### 4.7. Quantitative Real-Time PCR Validation

The reverse transcriptase (RT) reaction was performed by using RT reaction mix (Bio-Rad, Hercules, CA, USA) and oligo (dT) random hexamers. The reaction was performed at 37 °C for 1 h, followed by 42 °C for 1 h. PCR amplification was performed using SYBR Green Universal Master Mix. Briefly, repeated reactions were performed with 1 μL of template cDNA, 2 × Universal Master Mix and 100 nM primers (final volume 12.5 μL) and analyzed in 96-well reaction plates (Bio-Rad, Hercules, CA, USA). All samples were normalized to β-actin, and the experiment was repeated 3 times.

### 4.8. GO and KEGG Pathway Analysis

GO analysis (http://www.geneontology.org, accessed on 8 November 2018) was used to characterize properties of genes and gene products, including molecular functions, cellular components, and biological processes. GO classifies genes hierarchically, revealing gene regulatory networks based on biological processes. Predicting major pathways of differentially expressed genes using KEGG mapping.

### 4.9. Analysis of the LncRNA-mRNA Co-Expression Network

According to the correlation between the differentially expressed mRNAs and LncRNAs, the co-expression network of LncRNA-mRNA was constructed according to the normalized signal intensities of specific expression levels of mRNAs and LncRNAs by Cytoscape software (The Cytoscape Consortium, San Diego, CA, USA). Coding genes of the “necroptosis” pathway and the LncRNAs were identified by Pearson’s correlation coefficients, equal to or greater than 0.9.

### 4.10. RAW264.7 Cell Transfection

In brief, 1 × 10^6^ RAW264.7 cells were seeded in 6-well plates. ZETA LIFE Advanced (Zeta-Life, San Francisco, CA, USA) and siRNA duplexes were mixed while the plates were shaken gently. Three various siRNA-NR_003508 (GenePharma, Shanghai, China) were used, and the sequences of the siRNAs are listed below:

#150: Sense (5′-3′): GCGUUGAUUCAGUCAACUUTT

Antisense (5′-3′): AAGUUGACUGAAUCAACGCTT

#1520: Sense (5′-3′): GCACACGGUCACUGAAAUUTT

Antisense (5′-3′): AAUUUCAGUGACCGUGUGCTT

#2345: Sense (5′-3′): ACACUGCUAGAAAUAAAUUTT

Antisense (5′-3′): AAUUUAUUUCUAGCAGUGUGC

### 4.11. Immunoblotting Analysis

To validate the results of LncRNA on genes in the necroptosis pathway, we constructed siRNA for NR_003508 and then transfected siRNA-control, siRNA for NR_003508 (si-NR), LPS (1 µg/mL), and siRNA for NR_003508 (si-NR) + LPS (1 µg/mL) into mice RAW264.7 cells for 12 h by Zeta Life transfection reagent (Zeta-Life, San Francisco, CA, USA), respectively. Subsequently, we extracted all the above cells protein following the Western blot protocol and detected RIPK3, RIPK1, and MLKL expression by using specific antibodies against RIPK3, RIPK1, MLKL, and β-actin with appropriate peroxidase-labeled secondary antibodies. The blots were then developed using the enhanced chemiluminescence reagent (Amersham Biosciences, Piscataway, NJ, USA). All above antibodies were from Abcam(Cambridge, UK), except the antibodies against β-actin, which were products of protein tech (protein tech, Chicago, IL, USA).

### 4.12. Immunofluorescence

RAW264.7 cells were rinsed with 1 × PBS, fixed with 4% paraformaldehyde for 20 min, permeabilized with 0.1% Triton X-100 for 20 min, blocked with 3% BSA for 1 h, and incubated with primary antibodies at 37 °C for 3 h. The primary anti-p-MLKL antibody (Affinity, Cincinnati, OH, USA) was used at a 1:100 dilution that was diluted using 3% BSA. Secondary antibodies (goat antibodies conjugated to Alexa Fluor 488) were diluted in 1 × PBS and incubated for 1 h at RT. The slides were then washed with 1 × PBS three times and mounted with DAPI-containing mounting medium (ZSGB-BIO, Beijing, China). Images were captured by confocal microscopy (LEICA, Wetzlar, Germany).

### 4.13. PI Staining

The RAW264.7 cells were seeded (1 × 10^5^ cells/mL) and grown on covered, glass-bottom dishes for 12 h. After treatment, the cells were incubated with the indicated PI dye at 37 °C for 15  min and washed with PBS. Subsequently, the labeled cells were photographed using a laser confocal microscope (LEICA, Wetzlar, Germany). At least 10 cells from each experiment were chosen randomly and analyzed with Image J.

### 4.14. Statistical Analysis

Statistical evaluation of the data was performed by one-way ANOVA for comparisons of differences between the two groups. A value * *p* < 0.05 represent a statistical difference and a value ** *p* < 0.01 and *** *p* < 0.001 set to represent a statistically significant difference. Data were presented as the mean ± standard deviations (SD). All the data collected were obtained from at least three independent experiments for each condition.

## 5. Conclusions

In the present study, we examined the expression of mRNAs and LncRNAs in ARDS by microarray analyses, verified their expression, and confirmed the function of DE LncRNA on ARDS via regulating genes expression in the necroptosis signal pathway. This will be a novel insight for revealing the pathogenesis mechanism of ARDS.

## Data Availability

All the data and material are available in the manuscript.

## Supplementary Materials

The following are available online at https://www.mdpi.com/article/10.3390/pathogens11050532/s1. Table S1: List of differentially expressed lncRNAs compared with control. Table S2: List of differentially expressed mRNAs compared with control. Table S3: The reproducibility of LncRNA-mRNA co-expression network compared with other’s study.
